# Shining a spotlight on the inclusion of disabled participants in clinical trials: a mixed methods study

**DOI:** 10.1186/s13063-024-08108-7

**Published:** 2024-04-26

**Authors:** Yoshiko Sakuma, Marie L. E. Miller, Daphne S. Babalis, Alex Baker, Meena Reddi, Aisha Anjum, Jane Bruton, Kathryn N Jones, Umm Zeinab Mulla, Henock Taddese

**Affiliations:** 1 Faculty of Medicine, School of Public Health, Imperial College London, South Kensington Campus, London, SW7 2AZ UK; 2 Imperial Clinical Trials Unit, School of Public Health, Imperial College London, Stadium House, 68 Wood Ln, London, W12 7RH UK; 3 Imperial Clinical Trials Unit - Cancer, Department of Surgery and Cancer, Convergence Science Centre, Imperial College London, South Kensington Campus, London, Cancer Research SW7 2AZ UK; 4 Patient Experience Research Centre, Imperial College London, South Kensington Campus, London, SW7 2AZ UK; 5 Faculty of Medicine, School of Public Health, Medical School, Imperial College London, St Mary’s Campus, 167, Praed Street, London, W2 1NY UK

**Keywords:** Clinical trials, Mixed methods study, Under-served groups, Disability, Disabled people, Carers, Inclusion, Exclusion, Equality, Diversity

## Abstract

**Background:**

It is crucial to include a wide range of the population in clinical trials for the outcome to be applicable in real-world settings. Existing literature indicates that under-served groups, including disabled people, have been excluded from participating in clinical trials without justification. Exclusion from clinical trials exacerbates disparities in healthcare and diminishes the benefits for excluded populations. Therefore, this study was conducted to investigate potential obstacles that prevent disabled people from participating in clinical trials in the United Kingdom (UK).

**Methods:**

The study was carried out through an explanatory sequential mixed methods design. The Imperial Clinical Trials Unit devised and implemented an online questionnaire-based survey (with open/closed-ended questions) and an online focus group discussion. The target population were disabled people, family members/carers of disabled people and staff involved in clinical trials, whereupon the sample was recruited by convenience sampling methods via posters and emails through various networks. The Qualtrics XM survey system was used as the host platform for the online survey, and Microsoft Teams was used for an online focus group discussion. The focus group discussion was conducted to gain a deeper understanding of the themes identified from the survey responses. We analysed responses to the survey via descriptive analysis and used thematic analysis to synthesise the free-text answers from the survey and focus group discussion.

**Results:**

We received 45 responses to the survey questionnaire and 5 disabled people took part in a focus group discussion. Our findings highlighted the differences between the perspectives of researchers and those “being researched” and different types of barriers experienced by disabled people: opportunity barriers (inadequate recruitment strategy and ambiguous eligibility criteria), awareness barriers (perception of disability) and acceptance/refusal barriers (available support and adjustment, and sharing of trial results).

**Conclusion:**

Our findings support perspectives drawn from the Ford Framework regarding the need to consider all barriers, not just up to the point of enrolment into trials but also beyond the point of inclusion in clinical trials. We support calls for the introduction of legislation on including disabled people in clinical trials, implementation of industry/community-wide participatory approaches and the development of guidelines, a combined public–private approach.

**Supplementary Information:**

The online version contains supplementary material available at 10.1186/s13063-024-08108-7.

## Background

Clinical trials are conducted to assess the clinical effectiveness and safety of medical, surgical or behavioural interventions [1]. The efficacy and safety of interventions are influenced by intrinsic/extrinsic factors such as gender, race, age, ethnicity, medical history and genetic background. Outcomes may widely vary between populations [2]. Therefore, clinical trials should include a wide range of populations to enhance their generalisability [3].

However, research has highlighted that specific demographic groups have been excluded from clinical trials, and such exclusions would limit the generalisability of the results of clinical trials to real-world practice [4–11]. The population groups predominantly excluded from clinical trials, collectively referred to as “under-served groups”, include disabled people [12]. Such exclusions might be because of the lack of cultural competency of under-served groups, and better evidence that differences in genetics, implied by gender and ethnicity, significantly impact the response to interventions tested in clinical trials [13, 14].

This long-standing lack of inclusion of under-served groups in clinical trials risks widening health inequalities in the population and increasing the number of people left out of the benefits of healthcare advancement [15–21]. To date, disabled people have faced many obstacles that prevent them from participating in clinical trials. These barriers encompass a spectrum of issues, from the physical inaccessibility of clinical facilities, informed consent process, lack of disability awareness among healthcare professionals, and lack of clarity and justification on eligibility criteria [22–24]. It is especially worth noting the paradoxical scenario where disabled individuals may be excluded from clinical trials in research focusing on conditions that could lead to disability, such as neurological disorders [23, 24]. These multifaceted barriers underscore the need for comprehensive strategies to enhance accessibility and equity in clinical trial participation.

In response to this issue within the UK, in 2017, the National Institute for Health and Care Research (NIHR) launched the NIHR-INCLUDE initiative, which aims to bring a paradigm shift in attitudes surrounding inclusion in clinical trials [25]. Furthermore, in 2020, NIHR updated the guidance for applicants on Equality, Diversity and Inclusion (EDI) for study participants [26]. The new statement adds emphasis that all eligible participants should be offered the same opportunity for participate in clinical trials, regardless of geographical location, age, disability, gender reassignment, marriage and civil partnership, pregnancy and maternity, ethnicity, religion or belief, sex, sexual orientation, socioeconomic status, or access to health or social care [27]. In 2022, the NIHR also developed the INCLUDE Impaired Capacity to Consent Framework. This initiative is designed to enable the inclusion of individuals with impaired capacity due to conditions like dementia, stroke or learning disabilities to consent to clinical research [28]. 

Despite all the efforts made by NIHR towards inclusiveness and integration of disabled people into clinical trials in the UK [25, 26, 28], there are still no comprehensive guidelines for researchers to take into account various types of disability. This reflects a significant policy gap as disabled people account for 15% of the global population [29], 10.4 million people in the UK [30] and are more likely to have unmet medical needs compared to non-disabled people [31]. Although the challenges faced by under-served groups participating in clinical trials have been investigated internationally, research focusing on disabled people in this context is still limited. Most of the evidence currently available is from researcher perspectives, literature reviews or primary quantitative studies [22–24, 32–34]. The perspectives of carers and disabled people themselves are rarely represented in the research outputs.

In light of the above, this study aimed to explore the potential obstacles faced by people with various disabilities in participating in clinical trials. The study provides further insight into the inclusion of disabled people in clinical trials, as well as improving accessibility to clinical trials in the UK.

## Methods

### Theoretical framework

**Fig. 1:**
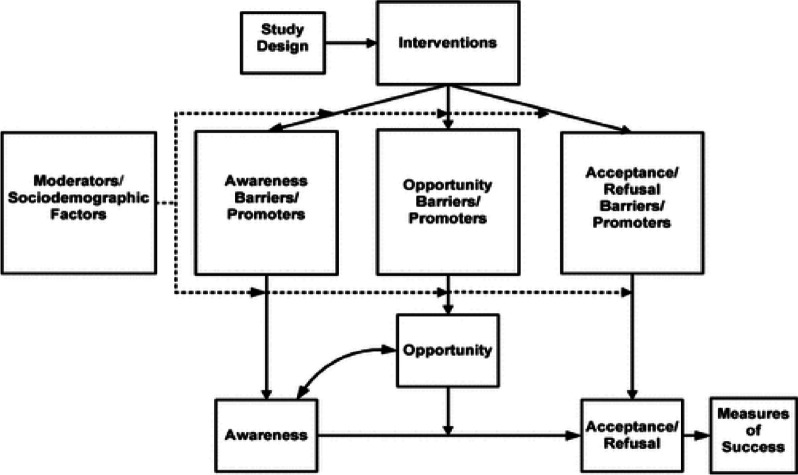
The conceptual framework categorises barriers to participate in clinical trials [35] (source: Jean G Ford. 2007. p.229)

We adopted a guiding conceptual framework, the Ford framework, developed by Jean G. Ford et al. [35]. This framework categorises barriers to participate in clinical trials based on their predicted effects on awareness, opportunity and the acceptance/refusal of participation. We used the Ford framework to guide the development of the online survey, topic guide and analysis of results (Fig. 1).

### Study design

**Fig. 2:**
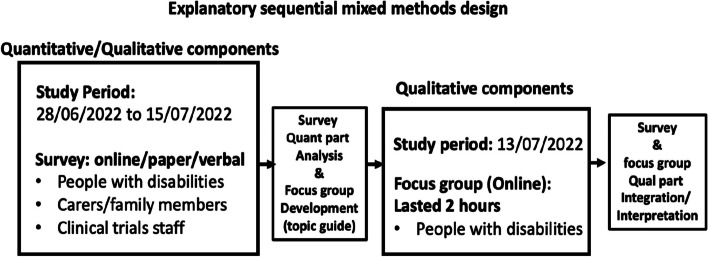
Explanatory sequential mixed methods design adopted in this study [36]

We employed an explanatory sequential mixed methods design (see Fig. 2) to generate a deeper understanding of the potential obstacles that disabled people may face when participating in clinical trials in the UK [36, 37]. An online survey with open- and close-ended questions was followed by an online focus group discussion devised and implemented by the Imperial Clinical Trials Unit (ICTU).

The Qualtrics XM survey system was the host platform for the online surveys and was open for approximately 2 weeks, from 28th June, 2022, to 15th July, 2022. The surveys consisted of multiple choice and free-text questions. The survey questions for disabled people and carers/families covered aspects of lived experiences of being disabled, experiences of participating in clinical trials and barriers and enablers to trial participation. The questions for trial staff were comprised of understanding of disability, clinical trials administration for disabled people and barriers and enablers for participation. The survey was limited to one completion per person with Qualtrics’s prevent multiple submission function, but carers/family members who cared for more than one person were encouraged to complete the survey more than once.

The consent question was included at the start of the survey form, and participants could not begin the survey unless they fully understood the purpose of the study and agreed to participate. If participants wished to participate in the focus group discussion, they were asked to contact the ICTU research team at the end of the survey. The participants received an invitation text and a participant information sheet detailing the focus group, which was sent by the project team. Additionally, they were requested to complete a consent form and a registration form to authorise the use of their data and to confirm their participation.

The preliminary survey results were used to elaborate and revise the topic guide (Additional file 1). The topic guide explored different factors based on the Ford framework. The focus group was conducted on Microsoft Teams and lasted approximately 2 h, including a break. The focus group participants were nominally reimbursed for their time and the Internet connection.

### Participant recruitment and selection

We followed convenience sampling to recruit participants for this study. The target populations were disabled people, carers/family members of disabled people and clinical trial staff. The adverts were distributed electronically and in paper via various distribution networks (Additional file 2). The inclusion and exclusion criteria of the study were:

#### Inclusion criteria

(A) A person with long-term disabilities and willing to disclose their disabilities.
(B) Carer/family member of a disabled person—willing to discuss the disability/relevant medical history of the person they care for.
(C) Staff involved in clinical trials—irrespective of their position/role, at any stage.

#### Exclusion criteria

(A) Disabled people and their carers/family members unwilling to disclose their disability.
(B) Non-disabled persons.

### Data analysis

All data and results were analysed and presented in accordance with the Good Reporting of a Mixed Methods Study (GRAMMS) guidance [38]. The quantitative data from the survey was descriptively analysed first. The qualitative data from the survey and focus group were thematically analysed guided by the Ford framework [35–41]. At the end of the analysis, the findings from the open-ended questions in the survey and focus group data were integrated to aid interpretation.

### Public involvement

According to the Ladder of Citizen Participation proposed by Arnstein [42], the study centred on partnerships and recruited five public partners with a range of disabilities, and one person who previously partnered with the ICTU. Public partners reviewed the participant information sheet, survey questions, images used in the surveys and materials for disseminating results, providing recommendations to enhance inclusivity. The recommendations included incorporating a text-to-read function into the survey, using images, providing videos with subtitles and increasing the text size.

### Researcher’s positionality

The study was conducted, analysed and integrated by an international student studying for a Master of Public Health at Imperial College London. The student is Asian, has a healthcare background and has experience conducting research with disabled people. The philosophical position adopted in the study, namely pragmatism, guided the project to utilise quantitative and qualitative findings to answer questions instrumental to the inclusion of disabled people in clinical trials.

### Available support/adjustment

To make the survey as accessible as possible, the study adverts informed participants that they could complete the survey online, paper or verbally if they wished by contacting the project team by email or telephone. All documents used in the study were compliant with the university accessibility guidelines. In addition, for the focus group, if participants were unfamiliar with Microsoft Teams, they were offered guidance beforehand. The results were developed in multiple formats, including a written report with lay terms, infographics and a video (with subtitles).

### Research team training

In this study, facilitators and staff involved in the focus group had completed safeguarding training on vulnerable people, Good Clinical Practice training, and underwent a Disclosure and Barring Service check.

## Results

### Demographic characteristics

A total of 45 people completed the survey. Of these, 26 (58%) were disabled people, 8 (18%) were carers/family members and 11 (24%) were trial staff. To tabulate results, numeric characters were assigned for disabled people (Disabled person 1–26), carers (Carer 1–Carer 8) and clinical trial staff (Staff 1–Staff 11). A focus group was conducted with five disabled people. To ensure anonymity, participants were assigned random alphanumeric characters (Participant 1–Participant 5). Table 1 explains the number of responses we received and the detailed characteristics of the survey respondents. Detailed demographic characteristics of focus group participants are available in Additional File 3.

**Table 1:** Numbers of survey responses received

	**Number (%)**
**Participant group (*n* = 45)**	
Person with disabilities	26 (58)
Carer/family member	8 (18)
Clinical trials staff	11 (24)
**Role of carer/family member (*n* = 8)**	
Family member	7 (88)
Paid carer	1 (12)
**Role of trials staff (*n* = 11)**	
Statistician	1 (9)
Trial manager/Coordinator	1 (9)
Research Nurse/Practitioner	6 (55)
Sponsor	3 (27)
**Types of disability (multiple choice)**	
Mobility	15 (26)
Mental	13 (22)
Vision	2 (4)
Hearing	3 (5)
Neurodiverse	6 (11)
Learning	5 (9)
Breathing	6 (11)
Others	7 (12)
**Number of disabilities (*n* = 34)**	
One	7 (21)
Two or more	27 (79)

The following sections discuss the results from the survey and focus group in an integrated manner. Tables 2 and Table 3 provide the summary statistics derived from the survey’s closed-ended questions. The results for each of these elements are elaborated upon in the following section. Additional file 4 provides supplementary information on the frequencies of themes derived from the focus group discussion.

**Table 2:** Descriptive results from survey multiple choice questions (disabled people and carers/family members)

Disabled people and carers/family members	Number (%)
**Have you ever taken part in clinical trials? (*n* = 34)**	
Yes	19 (56)
No	11 (32)
Did not answer	4 (12)
**Was the advert accessible and easy to understand? (*n* = 34)**	
Yes	9 (26)
No	2 (6)
To some extent	14 (41)
Can’t remember	3 (9)
Did not answer	6 (18)
**Impact of disability on day-to-day tasks (*n* = 34)**	
All the time	9 (26)
Most of time	16 (47)
Some of the time	7 (21)
Rarely	1 (3)
Not at all	1 (3)
**Impact of disability on communication (multiple answers)**	
Does not affect my communication	11 (12)
Vision	2 (2)
Speech	3 (3)
Hearing	6 (6)
Writing	8 (8)
Reading	6 (6)
Memory	14 (15)
Understanding	13 (14)
Focusing	15 (16)
Planning	13 (14)
Other	4 (4)
**Was the building accessible? (*n* = 19)**	
Yes	13 (38)
No	0 (0)
To some extent	6 (18)
Can’t remember	0 (0)
**Were you asked if you needed any support? (*n* = 19)**	
Yes	8 (42)
No	4 (21)
Can’t remember	3 (16)
Did not answer	4 (21)
**Were you informed of the results? (*n* = 19)**	
Yes	7 (37)
No	10 (53)
Did not answer	2 (10)

**Table 3:** Descriptive results from survey multiple choice questions (clinical trials staff)

Clinical trials staff (*n* = 11)	Number (%)
**Are adverts for clinical trials in easily accessible places?**	
Yes	7 (64)
No	4 (36)
**Is your hospital or clinic easily accessible to disabled people?**	
Yes	6 (55)
No	5 (45)
**Are the participant recruitment rooms accessible to disabled people?**	
Yes	6 (55)
No	5 (45)
**Are certain disabled groups excluded from clinical trials?**	
Yes	7 (64)
No	4 (36)
**Are the trial assessments or visits too burdensome for disabled people?**	
Yes	9 (82)
No	2 (18)
**Disabled people being excluded unnecessarily due to ambiguous eligibility criteria?**	
Yes	9 (82)
No	2 (18)

#### Theme 1: Opportunity barrier—inadequate recruitment strategy and ambiguous eligibility criteria

- Inadequate recruitment strategy

##### Survey closed-ended questions

There were gaps in participants’ and researchers’ awareness and perceptions regarding clinical trial recruitment. The survey results show that 47% of disabled people and carers/family members said that advertisements were not fully accessible and understandable. In contrast, 55% of the staff involved in clinical trials reported that the trial venue was accessible to participants, while a higher percentage, 64%, reported that the advertisement for the trial call was accessible to participants.

##### Survey open-ended questions

A response from one disabled person pointed out that the current recruitment strategy for clinical trials is predominantly led by clinicians. This approach tends to limit inclusivity for people outside of the clinic, as the target population is often too selectively chosen. To reach disabled people, different recruitment tactics should be employed, such as approaching local communities, local media and pharmacies and using relatable promotional materials. For example, disabled people described what they thought would be an effective approach for enhancing recruitment.

“Use ‘real’ case examples of disabled people with different impairments and ‘conditions’ to recruit” [Disabled person 19].

##### Focus group discussion

Focus group participants highlighted how information tends to be less accessible to disabled people. They mentioned that clinical trial participants are mainly recruited directly from clinics or hospitals and trials are often not openly advertised. They underscored a need for broader access to information on how people can participate and which clinical trials are currently ongoing. Additionally, they highlighted that disabled people are more likely to be overlooked as part of the target audience for clinical trials.

(b) Ambiguous eligibility criteria

##### Survey closed-ended questions

Sixty-four of staff perceived that certain groups of disabled people are excluded from participating in clinical trials. Eight-two percent of staff indicated that eligibility criteria for disabled people are sometimes ambiguous and lead disabled people to be unnecessarily excluded from participating in clinical trials.

##### Survey open-ended questions

It was reported, mainly by clinical trial staff, that the eligibility criteria for disabled people largely depend on the discretion of the principal investigators and co-investigators. Clinical trial staff who responded to the survey questionnaire described the eligibility criteria of many clinical trials as a “grey area” for recruiting disabled people.

“This is ambiguous and leaves it up to the Investigator to interpret, they may be over cautious and exclude people ‘to be on the safe side’ when in fact these people are eligible” [Staff 2].

To improve this, respondents suggested that tailored inclusion and exclusion criteria for disabled people should be used, and disability and capacity to consent should be assessed at the individual level. However, it was also mentioned that implementing such adjustments takes time and financial consideration.

“To allow for patients with fluctuating capacity; however, they may then not be able to give informed consent if it is a more complex trial. The patients would have to be assessed individually to ensure they can retain and understand the information to give consent” [Staff 3].

##### Focus group discussion

The focus group highlighted that the current eligibility criteria of clinical trial designs are not grounded in “realistic expectations” of disabled people. Disabled people also mentioned that researchers feared that by including a small number of disabled people would hamper statistics and potentially introduce confounding factors in the model.

“Funders, e.g., NIHR, need to recognise the requirements for inclusivity can be unrealistic” [Participant 2].

#### Theme 2: Awareness barrier—perception of disabilities

##### Survey closed-ended questions

Ninety-four percent of disabled people and carers/family members stated that their disability interfered with their activities of daily living, and 88% of them said it affected their communication. Of these, 26% of disabled people and carers/family members said their daily tasks were always affected.

##### Survey open-ended questions

Disabled people who responded to the open-ended questions in the survey stated that the perception of disability that clinical trial staff have remains superficial and sometimes over-medicalised, thereby highlighting the need to understand better how having a disability affects peoples’ daily lives in different ways. It is often challenging to fully understand the extent of disability, particularly in individuals with invisible disabilities (conditions that are not immediately obvious, e.g., chronic pain, hearing loss, mental health conditions). These individuals run the risk of not being perceived as disabled.

“People think that I’m lazy, that losing weight and exercise would solve all my problems. They do not understand the pain with every single task” [Disabled person 10].

##### Focus group discussion

In addition to the survey respondents, focus group participants highlighted that invisible disabilities and multiple conditions are often not recognised in the research. Participant 5 described the importance of invisible and multiple disabilities being recognised, along with her experience of having a visual impairment but not being recognised by others as having a disability. Thus, understanding different dimensions of disability by staff and sponsors involved in clinical trials is essential; this also relates to whether support and adjustments are available to disabled people.

“Visual impairments fall off the radar, disabilities aren’t necessarily declared. And having multiple conditions is not always recognised in surveys or trials” [Participant 5].

#### Theme 3: Acceptance/refusal barrier—available support and adjustment, sharing results

- Available support and adjustment

##### Survey closed-ended questions

Although 82% of staff were aware that the assessments and site visits could be burdensome for disabled people, only 42% of disabled people had been asked about the need for support/adjustment when participating in clinical trials.

##### Survey open-ended questions

Survey respondents highlighted that having a disability had several consequences in the lives of disabled people and their participation in clinical trials — these burdens on disabled people include time and financial, physical and mental constraints. Twenty-one percent of survey respondents stated that they were not asked in advance about the support and help they would need when taking part. Although the need for support is recognised among staff, staff responses revealed that this is subject to resources and financial constraints, such as whether funders are willing to support such as sign language interpreters and cover travel expenses.

“For me, length of travel, accessibility to trial rooms, and how long the trial might take would all be factors, and I understand these factors may well be outside the researchers’ scope” [Disabled person 12].

##### Focus group discussion

Similarly, disabled people who participated in the focus group discussed the need for providers to recognise that participating in clinical trials, in addition to their usual care and treatment, is not easy and that they would need to make considerable preparation to participate, such as transport, whether the venue clinic has soft seating, and whether there are places for refreshments and breaks. They highlighted that support and adjustment for participation in clinical trials need to reflect the realities and needs of people with disabilities more accurately.

“I would like to ‘do my bit’, I might be in a unique position because of my disability but also can’t do much because of my disability” [Participant 4].

“It is hard work, you always have to plan, plan, plan. If I need to go to the shops, how far is it, can I get there, will I need the bathroom. All these worries make it difficult before you even leave the house” [Disabled person 5].

(b) Sharing results

##### Survey closed-ended questions

Interesting data on the sharing of clinical trial results were obtained. Out of the 19 participants in the study who indicated they had participated in clinical trials, only seven (37%) of the carers/family members and disabled individuals were informed about the trial results.

##### Survey open-ended questions

The clinical trial staff did not raise any issues regarding sharing results and content. However, the disabled people and carers who responded to the questionnaire highlighted this issue. The following quote stated that the results were unacceptable as the participants felt they had been misrepresented. This highlights the unpicked issue of “how” and “when” results should be shared with disabled people/carers.

“I received information on the study results but not before I had read about it in the national press, and the way that the results were presented made me feel that I had been an inadequate mother….” [Carer 8].

## Discussion

This study explored potential obstacles that influence the participation of disabled people in clinical trials in the UK from the perspective of disabled people, carers and clinical trial staff, using an explanatory sequential mixed methods design. Guided by The Ford framework [35], we specified multiple factors across different dimensions: “opportunity barriers” (inadequate recruitment strategy, ambiguous eligibility criteria), “awareness barriers” (perception of disability) and “acceptance/refusal barriers” (available support and adjustment, sharing results). There was a general sentiment among all participant groups that disabled people are often unnecessarily excluded from participating in clinical trials. Additionally, we note key differences between the “being researched” and the “researcher” perspectives and, specifically, highlight a key topic that had not been signalled by the guiding framework: issues/problems related to “sharing of clinical trial findings”.

The most frequently reported barrier in this study was related to the opportunity to participate, such as inadequate recruitment strategies and ambiguous eligibility criteria for clinical trial participation. Opportunities for recruitment into clinical trials with disabled people are very limited; there is a strong desire for more openly publicised advertising and recruitment methods. This can be improved by using public involvement with various public partners with lived experience of disability at all stages of clinical trials [43].

Another barrier to participation is the presence of ambiguous eligibility criteria, such as the lack of clearly justified criteria for individuals with conditions that may put participants at risk during the trial. This ambiguity may not adequately account for the diverse range of disabilities among participants in clinical trials [44]. This has been discussed in many previous studies and is a very complex topic that has not yet reached a consensus on the implementation of standardised practice [45–47]. However, the very high rate of ambiguous exclusion criteria and inadequate exclusion of persons with disabilities in clinical trials has been highlighted by Camanni et al. [44]; their study revealed that, in 44.5% of the trials, the discretion of the investigators regarding the exclusion of study participants was considered implicit exclusion criteria [44]. Although some studies have proposed the consideration of alternative methods, such as proxy consent or the use of disability assessment tools at the individual level, there are still only a few reports of their actual implementation in clinical trials [48–50]. No clear measures on this topic have yet been presented, however, employing a tool like the Impaired Capacity to Consent Framework, explicitly designed for individuals with impaired capacity to consent, could be implemented to assist researchers in determining the necessary actions and resources, potentially guiding interventions for patients with diverse disabilities [28].

The importance of the perception of disability, identified as an awareness barrier, has also been discussed by Marjanovic et al. [51]. Lack of appropriate knowledge and awareness among stakeholders affects the quality of trial outcomes and retention of participants [51]. Stakeholders in clinical trials should recognise that participants may have visible as well as invisible disabilities and seek to understand how their disabilities impact their lives and what support they need.

We also underscore the importance of readily available help and adjustments, which are key elements for the acceptability of participation. We recommend training staff and incorporating planning and guidance into the Standard Operating Procedure guidelines for centres running clinical trials, as well as considering the physical, mental, financial and time constraints of trial participants. In this study, disabled people and their carers said they would have been able to participate in clinical trials if they had received appropriate support, which is in line with the findings of a previous study by Feldman et al. [52]. Our findings reaffirm the need for better awareness regarding critical support and adjustment needs.

The one theme identified in this study that did not fall in the Ford framework was the factor related to sharing clinical trial results. In clinical trial participation, it should be an ethical norm for participants to receive ongoing progress updates and study results, which are significant aspects of clinical trial inclusion [53]. The importance and effectiveness of sharing the results of clinical trials, as well as approaches to sharing results, have been described in prior literature [54]. This practice can increase potential participation in future clinical trials and foster trust in research [54]. The specific needs of people with disabilities and carers, obtained in this study (e.g., appropriate language, timing, methods), add future insight concerns regarding this crucial step in the research process. However, there is still a notable gap in the evidence regarding the best practices for sharing research results with people with disabilities and their carers, particularly concerning the optimal timing, mode and content of such communication. It is clear that more detailed research is required to address this evidence gap.

## Strengths and limitations

This study adds valuable evidence to the topic with actual voices from disabled people, carers/families and trial staff. This study was conducted in partnership with public partners with disabilities at different stages of the research. This process has helped make the study more inclusive [55]. In addition, the study was designed to be accessible to as many participants as possible, whereby we offered a range of survey formats to accommodate the different preferences of survey respondents.

However, in this study, the participants were recruited without specific criteria regarding the types/degree of disability. Therefore, the present study cannot comprehensively consider all disabilities. Second, due to time and resource constraints, we were not able to include carers/families and trial staff in focus groups nor conduct semi-structured interviews. To some extent, we have overcome this limitation by combining qualitative data from the survey with findings from the focus group. Third, this study was unable to collect sociodemographic information in the online questionnaire. Disability status has intersectionality with other sociodemographic backgrounds such as gender, age, sexuality and ethnicity. Future studies should collect disability status as sociodemographic information and conduct sub-analyses to provide additional insights into the inclusion of other under-served population groups.

## Conclusion

We recommend the introduction of legislation on the inclusion of under-served groups in clinical trials, the implementation of an industry/community-wide participatory approach, such as guidance or training for stakeholders, and the development of guidelines specific to disability. These measures will go a long way towards ensuring optimal participation of disabled people and other under-served populations and enhance the validity and generalisability of clinical trial results.

### Supplementary Information

**Additional file 1.** Topic guide. Questions were used in focus group.

**Additional file 2.** Survey distribution network. Table of dissemination network for the project.

**Additional file 3.** Demographic characteristics of focus group participants. Table of demographic characteristics of focus group participants.

**Additional file 4.** Frequency of identified themes. Frequency table of thematic analysis.

**Additional file 5.** Survey for disabled people. Survey questions for disabled people.

**Additional file 6.** Survey for carers/family members. Survey questions for carers/family members.

**Additional file 7.** Survey for trial staff. Survey questions for trial staff.

**Additional file 8.** Good Reporting of A Mixed Methods Study (GRAMMS) Guidelines.

## Data Availability

Other data is available upon reasonable request.

## Abbreviations

BRC: Biomedical Research Centre
EDI: Equality, Diversity and Inclusion
ICTU: The Imperial Clinical Trials Unit
NIHR: National Institute for Health and Care Research
NIHR-INCLUDE: The NIHR-Innovations in Clinical Trial Design and Delivery for the Under-served
UK: United Kingdom
