# Supplementary material for: Shining a spotlight on the inclusion of disabled participants in clinical trials: a mixed methods study
Source: Trials. 2024 Apr 26;25:281. doi: 10.1186/s13063-024-08108-7 (PMC11046956; doi:10.1186/s13063-024-08108-7)
Supplement: Supplementary file 1 — Additional file 1. Topic guide. Questions were used in focus group. [file 13063_2024_8108_MOESM1_ESM.docx]

**Appendix 1. Topic guide**
