# Supplementary material for: Shining a spotlight on the inclusion of disabled participants in clinical trials: a mixed methods study
Source: Trials. 2024 Apr 26;25:281. doi: 10.1186/s13063-024-08108-7 (PMC11046956; doi:10.1186/s13063-024-08108-7)
Supplement: Supplementary file 2 — Additional file 2. Survey distribution network. Table of dissemination network for the project. [file 13063_2024_8108_MOESM2_ESM.docx]

**Appendix 2. Survey distribution network**

| **Survey for disabled people and carers/family members** |
| --- |
| Imperial Clinical Research Facility research volunteer’s database |
| Public partners who are involved in ICTU trials |
| Twitter (ICTU, Patient Experience Research Centre) |
| Voice global platform |
| Individual charities (RNIB, Deafblind, Carers.org, Scope, National Autistic Society) |
| **Survey for Clinical Trials Staff** |
| UK Trial Managers Network |
| UKCRC Clinical Trials Network |
| Twitter (ICTU, Patient Experience Research Centre) |
| Voice global platform |
| ICTU Staff email |
