# Supplementary material for: Shining a spotlight on the inclusion of disabled participants in clinical trials: a mixed methods study
Source: Trials. 2024 Apr 26;25:281. doi: 10.1186/s13063-024-08108-7 (PMC11046956; doi:10.1186/s13063-024-08108-7)
Supplement: Supplementary file 3 — Additional file 3. Demographic characteristics of focus group participants. Table of demographic characteristics of focus group participants. [file 13063_2024_8108_MOESM3_ESM.docx]

**Appendix 3. Demographic characteristics of focus group participants**

|  | **Number (%)** |
| --- | --- |
| **Age**  45-54  65-74 | 3 (60)  2 (40) |
| **Sex assigned at birth**  Male  Female | 2 (40)  3 (60) |
| **Ethnicity**  White  Asian (Pakistani) | 4 (80)  1 (20) |
| **Employment**  Unemployment  Retired | 2 (40)  3 (60) |
| **Experience of participating in clinical trials**  Yes  No | 3 (60)  2 (40) |
