# Supplementary material for: Shining a spotlight on the inclusion of disabled participants in clinical trials: a mixed methods study
Source: Trials. 2024 Apr 26;25:281. doi: 10.1186/s13063-024-08108-7 (PMC11046956; doi:10.1186/s13063-024-08108-7)
Supplement: Supplementary file 4 — Additional file 4. Frequency of identified themes. Frequency table of thematic analysis. [file 13063_2024_8108_MOESM4_ESM.docx]

**Appendix 4. Frequency of identified themes**

| **Theme** | **In survey sample**  **N=45 (%)** | **In FGD sample**  **N=5 (%)** |
| --- | --- | --- |
| **Opportunity Barrier**  Inadequate recruitment strategy  Ambiguous eligibility criteria | 25 (55)  15 (34) | 2 (38)  2 (43) |
| **Awareness Barrier**  Perception of disability | 42 (94) | 5 (90) |
| **Acceptance/Refusal Barrier**  Available support and adjustment  Sharing results | 37 (83)  38 (84) | 4 (70)  N/A |
