# Supplementary material for: Shining a spotlight on the inclusion of disabled participants in clinical trials: a mixed methods study
Source: Trials. 2024 Apr 26;25:281. doi: 10.1186/s13063-024-08108-7 (PMC11046956; doi:10.1186/s13063-024-08108-7)
Supplement: Supplementary file 6 — Additional file 6. Survey for carers/family members. Survey questions for carers/family members. [file 13063_2024_8108_MOESM6_ESM.docx]

**Learning how to improve access to clinical trials for people living with disability**

This survey is about how people with disabilities might be excluded from clinical trials. We are hoping to learn about how to include disabled people better in clinical trials by conducting this research and hoping to improve access to clinical trials for many people.

**This survey is for people who are either carers or close family members of a disabled person.**

By participating in this survey, you are agreeing that the information you provide can be used to guide how disabled people can be better represented in clinical trials. All information provided will be anonymous. Please do not enter any information that could identify you personally when answering the questions in this questionnaire. You can agree to be contacted for further research activities at the end of the survey but this is not linked to your survey response. Once submitted, your data cannot be withdrawn.

Your data will be held and processed in line with Imperial College London policies on GDPR and privacy. Further information can be found at <https://www.imperial.ac.uk/clinical-trials-unit/dataprotection/>

All questions, apart from what disabilities the person you care for has which is needed for our research), are optional to complete, if you do not feel comfortable in answering, you may skip to the next question.

If you have any queries or complaints, please contact

1. I confirm that I have read and understand the participant information sheet on <https://www.imperial.ac.uk/clinical-trials-unit/dataprotection/> (dated 16/05/2022 version 1.0) for the above study and have had the opportunity to ask questions which have been answered fully.
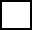

2. I understand that I am giving this consent based on what I believe would be my relative/ friend/ partner’s wishes. In my opinion they would be willing to participate.
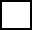

3. I understand that their participation is voluntary, and I or the person I am consenting for are free to withdraw up until the point at which the survey answers are submitted, without giving any reason and without our legal rights being affected.
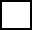

4. I agree that my relative / friend / partner’s consent will override my consent on their behalf if or when they are able to give informed consent themselves.
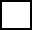

5. I agree to my relative/ friend / partner taking part in the study.
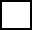


**What we mean by a clinical trial:**

- A treatment trial - compares the effects of 1 treatment with another. It may involve patients, healthy people, or both. A treatment could be a medication, an assistive device or a therapy, and may also be a combination of these.
- An observational trial - collecting your medical data and samples but not testing any treatments. Samples could include for example, blood samples or urine and faeces samples. Medical data could be recent blood tests results, recent scan results, questionnaires about your current health and quality of life.

**What we mean by disabilities** are any conditions that have a substantial and long term effect on the ability to carry out day-to-day activities including:

- Physical disabilities for example a spinal cord injury causing paralysis
- Hidden disabilities for example chronic fatigue
- Mental health conditions for example depression
- Neurodiverse conditions for example ADHD (Attention deficit hyperactivity disorder), Tourette’s, dyslexia
- Cognitive impairments for example dementia or Alzheimer's disease

Therefore, we ask all participants to identify the disability of your family member or the person you care for. If you do not wish to disclose their disability you will not be able to take part.

**About the person you care for**

1. Are you a carer or close family member of someone with a disability/ies?
   - Paid carer of a disabled person (with no family connection)
   - Close family member with specific care responsibilities
   - Close family member of a disabled person
   - None of the above

If none of the above - thank you for taking part in our survey. Unfortunately, as we are looking for information on how people with disabilities are excluded from clinical trials there will be no further questions for you to answer.

If you care for more than one person please complete a different survey for each person

1. What disability/ies does the person you care for have

For the other disability options, please write in full rather than initials

Select all that apply

| - Vision (e.g. blindness or partial sight)   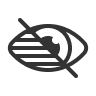 | - - Hearing (e.g. deafness or partial hearing)   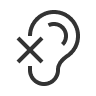 | - - Mobility (e.g. difficulty walking short distances, climbing stairs, lifting and carrying)   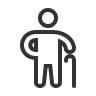 |
| --- | --- | --- |
| - - Learning, concentrating or remembering   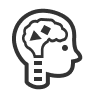 | - - Mental health   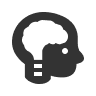 | - - Stamina or breathing difficulty   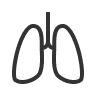 |
| - - Social or neurodiverse conditions (such as Autism, Attention Deficit Disorder or dyslexia)   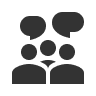 | - - Other impairment please describe   ________________ | - - Other impairment please describe   ________________ |
| - - Other impairment please describe   ________________ | - - Other impairment please describe   ________________ |  |

If none of the above - thank you for taking part in our survey. Unfortunately, as we are looking for information on how people with disabilities are excluded from clinical trials there will be no further questions for you to answer.

1. Does their disability/disabilities affect their ability to perform day-to-day tasks such as washing, cooking, going shopping, working etc?
   - Not at all
   - Rarely
   - Some of the time
   - Most of the time
   - All of the time
2. Does their disability/disabilities affect their communication due to difficulties with: Select all that apply

| - Does not affect their communication | - - Vision   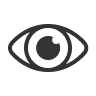 | - - Speech   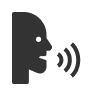 |
| --- | --- | --- |
| - - Hearing   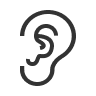 | - - Writing   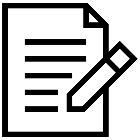 | - - Reading   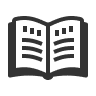 |
| - - Memory   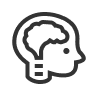 | - - Understanding   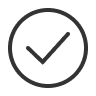 | - - Focusing   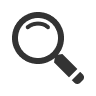 |
| - - Planning   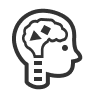 | - - Other, please describe   ________________ |  |

Please provide any further comments related to the question above

**Taking part in a trial**

1. Has the person you care for ever taken part in a clinical trial? A clinical trial usually compares the effects of 1 treatment with another. It may involve patients, healthy people, or both. A trial may also be an observational, collecting your medical data and samples but not testing any treatments.

Yes No

If Yes, please provide some information on the trial such as the condition, treatment studied etc

**If No, go to question 10**

1. If you, or the person you care for, saw the clinical trial advertised, was the advert accessible and easy to understand for the person you care for? please provide details
   - Yes ___________________________________
   - No ___________________________________
   - To some extent _________________________
   - can’t remember ___________________________
2. If the person you care for came in person to a clinic to agree to take part in a clinical trial, was the building accessible? Please consider this in relation to a physical disability as well as other disabilities for example signs around clinics, noise levels, lighting, greetings by staff when entering the clinic etc.
   - Yes ___________________________________
   - No ___________________________________
   - To some extent _________________________
   - can’t remember ___________________________
3. Was the information they were given about the trial accessible and easy to understand?
   - Yes ___________________________________
   - No ___________________________________
   - To some extent _________________________
   - can’t remember ___________________________
4. Were you asked if you needed any support to be able to take part in the clinical trial and attend the trial visits?

If yes, what support/adjustments were given?

Could anything have been done differently that would have improved your experience as a disabled participant?

1. If the person you care for has taken part in a trial, were you/they informed of the results?

Yes No

If so, did they understand these, were there options for different formats/assistance to understand? Was this suitable for the needs of their disability? Would you or they have preferred for this to have been done differently?

**Helping the person you care for to take part**

1. Would the person you care for take part in a clinical trial if they haven’t before or would they again, if they already have before?

Yes No

If yes is there anything researchers can do to improve your/their experience?

If no, what are the reasons they do not want to participate?

1. How would you like the trial information explained to help you and the person you care for understand it the most

Select all that apply

- - paper information sheet
  - Information Video
  - Information Video with subtitles
  - Diagrams/pictures/poster
  - A discussion with member of the medical team in person

1. What help/support/adjustments do you think you, as a carer and the person you care for, would need in order for the person you care for to participate? Consider that taking part in a clinical trial is a commitment on top of your current responsibilities as a carer.
2. What would put off the person you care for, participating in a clinical trial/make them want to drop out during the trial?
3. A trial can last anywhere between a few weeks to 10 years. What would help you/motivate you to assist the person you care for to be part of a trial until the end of the trial?

As part of this research, we are also conducting focus groups to gain further insight into how disabilities are excluded from clinical trials and how this could be improved going forward.

*A focus group is a group of people gathered together to discuss and provide feedback on a topic with the researchers.*

If you are interested in taking part in these groups, please contact the Imperial Clinical Trials Unit - Equality, Diversity and Inclusion project team at, and we will be in touch if we require your help.

By contacting us you are agreeing to share your contact email, telephone number or postal address with the team, in line with Imperial College London GDPR and privacy policies. More information can be found at <https://www.imperial.ac.uk/clinical-trials-unit/dataprotection/>

Thank you for participating in this survey
