# Supplementary material for: Shining a spotlight on the inclusion of disabled participants in clinical trials: a mixed methods study
Source: Trials. 2024 Apr 26;25:281. doi: 10.1186/s13063-024-08108-7 (PMC11046956; doi:10.1186/s13063-024-08108-7)
Supplement: Supplementary file 7 — Additional file 7. Survey for trial staff. Survey questions for trial staff. [file 13063_2024_8108_MOESM7_ESM.docx]

**Learning how to improve access to clinical trials for people living with disability**

This survey is about how people with disabilities might be excluded from clinical trials. We are hoping to learn about how to include disabled people better in clinical trials by conducting this research and hoping to improve access to clinical trials for many people.

By participating in this survey, you are agreeing that the information you provide can be used to guide how disabled people can be better represented in clinical trials. All information provided will be anonymous. Please do not enter any information that could identify you personally when answering the questions in this questionnaire. You can agree to be contacted for further research activities at the end of the survey but this is not linked to your survey response. Once submitted, your data cannot be withdrawn.

Your data will be held and processed in line with Imperial College London policies on GDPR and privacy. Further information can be found at <https://www.imperial.ac.uk/clinical-trials-unit/dataprotection/>

All questions are optional to complete, if you do not feel comfortable in answering, you may skip to the next question.

If you have any queries or complaints, please contact

1. I confirm that I have read and understand the participant information sheet at <https://www.imperial.ac.uk/clinical-trials-unit/dataprotection/> (version 1.0 dated 16/05/2022) for the above study and have had the opportunity to ask questions which have been answered fully
2. I understand that my participation is voluntary, and I am free to withdraw up until the point at which I submit the survey answers without giving any reason and without my legal rights being affected.
3. I consent to take part in the above study.
4. What is your role in clinical trials?
   - Chief Investigator
   - Principal Investigator
   - Research Nurse/Trial Practitioner
   - Trial Manager/Coordinator (sponsor)
   - Trial Manager/Coordinator (site)
   - Sponsor Representative
   - Statistician
   - Site support e.g. administration, data management, R&D, pharmacy
   - Other – please provide details

_____________________________________________

1. What is your general understanding of disability?

By disabilities we mean are any conditions that have a substantial and long term effect on the ability to carry out day-to-day activities including physical disabilities, as well as hidden disabilities and mental health disorders. Please answer all questions with this in mind.

1. What type of trials do you recruit to (tick all that apply)?
   - Healthy volunteer trials
   - IMP/treatment trials
   - Device trials
   - Observational studies
   - Translational studies
   - Other – please describe

_____________________________________________

1. Are the clinical trials you are involved with advertised to the public?
2. Are adverts for clinical trials in easily accessible places? Please provide details
   - Yes ___________________________________
   - No ___________________________________
3. Do adverts get reviewed by the public before being used? Please provide details
   - Yes ___________________________________
   - No ___________________________________
4. Is your hospital clinic easily accessible to disabled people? Consider not just physical disabilities but hidden disabilities and mental health disorders such as noise levels, signage, lighting, greetings by staff when entering the clinic etc

Yes No

If not why?

1. Are the participant recruitment rooms accessible to disabled people? Consider not just physical disabilities but hidden disabilities and mental health disorders

Yes No

*If not why?*

1. Is there support available for those with social/behavioural difficulties/mental health disorders, to take part in trials (for those that may have some limitations but may not necessarily lack capacity)
2. Clinical trial inclusion and exclusion criteria: are certain disabled groups excluded from clinical trials?

Yes No

If yes why?

1. What criteria could be removed or added to ensure greater inclusion of disabled participants?
2. Clinical trial design: are the trial assessments or visits too burdensome for disabled people preventing participation or retention to trials?

Yes No

If yes why?

1. Participant information resources: are these accessible to the whole disabled population? Consider not just physical disabilities but hidden disabilities and mental health disorders
2. Should a legal representative/consultee PIS and ICF for patients lacking capacity to consent be supplied as standard alongside a PIS/ICF directed to patients with capacity?
3. Should additional options for giving consent be provided such as e-consent, verbal consent to be used where appropriate? E-consent is a digital form of signing consent forms, this may be done in clinic on a tablet/computer or remotely via a link sent to patients. Verbal consent is the documentation in patient notes that consent was given verbally with a witness to the consent also recorded.
4. Do you think eligibility criteria can sometimes be ambiguous and lead to disabled people being excluded unnecessarily, for example ‘unable or unwilling to comply with the requirements of the study/protocol’?

Yes No

If yes why?

As part of this research, we are also conducting focus groups to gain further insight into how disabilities are excluded from clinical trials and how this could be improved going forward.

*A focus group is a group of people gathered together to discuss and provide feedback on a topic with the researchers.*

If you are interested in taking part in these groups, please email the Imperial Clinical Trials Unit - Equality, Diversity and Inclusion project team at, and we will be in touch if we require your help.

By contacting us you are agreeing to share your contact email, telephone number or postal address with the team, in line with Imperial College London GDPR and privacy policies. More information can be found at <https://www.imperial.ac.uk/clinical-trials-unit/dataprotection/>.

Thank you for participating in this survey
