# Supplementary material for: Shining a spotlight on the inclusion of disabled participants in clinical trials: a mixed methods study
Source: Trials. 2024 Apr 26;25:281. doi: 10.1186/s13063-024-08108-7 (PMC11046956; doi:10.1186/s13063-024-08108-7)
Supplement: Supplementary file 8 — Additional file 8. Good Reporting of A Mixed Methods Study (GRAMMS) Guidelines. [file 13063_2024_8108_MOESM8_ESM.docx]

**Good Reporting of A Mixed Methods Study (GRAMMS) Guidelines**

1. Describe the justification for using a mixed methods approach to the research question.

*Background section on page 5 to 6*

1. Describe the design in terms of the purpose, priority and sequence of methods.

*Methods section on page 7 to 8*

1. Describe each method in terms of sampling, data collection and analysis.

*Methods section on page 7 to 12*

1. Describe where integration has occurred, how it has occurred and who has participated in it.

*Methods section on page 9 to 10*

1. Describe any limitation of one method associated with the present of the other method.

*Discussion section on page 27-28.*

1. Describe any insights gained from mixing or integrating methods.

*Discussion section on page 24 to 28.*
